# What inhibits “speaking up” for patient safety among healthcare workers? A cross-sectional study in Malaysia

**DOI:** 10.1186/s12960-024-00916-x

**Published:** 2024-05-28

**Authors:** Alex Ren Jye Kim, Kimihiro Nishino, Mohamad Adam Bujang, Zubalqiah Zulkifli, Souphalak Inthaphatha, Eiko Yamamoto

**Affiliations:** 1 Department of Healthcare Administration, Nagoya University Graduate School of Medicine, 65 Tsurumai-Cho, Showa-Ku, Nagoya, 466-8550 Japan; 2 Quality and Training Unit, Sarawak General Hospital, Ministry of Health Malaysia, 93586 Kuching, Sarawak Malaysia; 3 Clinical Research Centre, Sarawak General Hospital, Ministry of Health Malaysia, 93586 Kuching, Sarawak Malaysia

**Keywords:** Speaking up, Withholding voices, Multiple types of healthcare workers, Patient safety, Malaysia

## Abstract

**Background:**

In healthcare, “speaking up” refers to when healthcare workers raise concerns regarding patient safety through questions, sharing information, or expressing their opinion to prevent harmful incidents and ensure patient safety. Conversely, withholding voice is an act of not raising concerns, which could be beneficial in certain situations. Factors associated with speaking up and withholding voices are not fully understood, especially in strong authoritarian societies, such as Malaysia. This study aimed to examine the factors associated with speaking up and withholding the voices of healthcare workers in Malaysia, thus providing suggestions that can be used in other countries facing similar patient safety challenges.

**Methods:**

This cross-sectional study was conducted in a tertiary hospital in Sarawak State, Malaysia. Data were collected from 474 healthcare workers from 43 departments using a self-administered questionnaire for speaking up and withholding voices measures in 4 weeks prior to data analysis as well as socio-demographic factors of healthcare workers (sex, age group, profession, department, weekly work hours for patient care, years of employment in the hospital, and the hierarchical level) and speaking up related climate of the working environment were recorded. Data were analyzed using descriptive statistics. Logistic regression was performed to find out (adjusted) odds ratio of frequent speaking up and withholding voices.

**Results:**

Nurse compared to doctors and healthcare workers with short weekly working hours were more likely to speak up. Healthcare workers in emergency and intensive care department, those with short years of employment, and those who worked at low hierarchical levels were less likely to speak up. Healthcare workers in discouraging environment towards speaking up were more likely to withhold their voices.

**Conclusions:**

This study demonstrates the characteristics of healthcare workers who speak up and those who withhold their voices in Malaysia. To ensure patient safety and prevent harm, it is essential to establish an encouraging environment that promotes speaking up and prevents withholding voices among healthcare worker, especially in circumstances where multiple types of healthcare workers with different socio-demographic backgrounds work together.

## Background

In the healthcare industry, the act of “speaking up” refers to when healthcare workers voice their concerns regarding patient safety. This can include issues such as non-compliance to standard operating procedures or poor clinical judgment and can be raised through questions, sharing information, or expressing their opinion to prevent harmful incidence and ensure patient safety [1–4]. A psychologically safe and encouraging environment promotes healthcare workers to speak up when they perceive patient safety concerns [4–6]. Psychological safety is a shared belief that healthcare workers can speak up with little or no fear of embarrassment, rejection, or punishment [4–6]. An encouraging environment for speaking up refers to a circumstance where healthcare workers observe others speaking up regarding safety concerns and are encouraged to speak up by their colleagues and supervisors [7–10]. The World Health Organization recognizes patient safety as an important topic and hence, selected the slogan “speak up for patient safety” for World Patient Safety Day in 2019 [11]. Speaking up is ethically essential for patient safety and has positive financial impact on the healthcare organization by avoiding harm and enabling cost savings [12]. Furthermore, speaking up regarding errors and near misses provides learning opportunities, which could prevent the recurrence of incidents, ultimately improving the healthcare system [12].

Withholding voice is the act of not raising concerns that could be beneficial in certain situations [7–10]. Barriers, such as sociocultural background and previous negative experiences of being disrespected or ignored, lead to withholding voice and even avoiding speaking up [1, 7–10, 13]. Ineffective communication or withholding voice contributed to 60–70% of incidents [14].

Despite the potential positive impact of speaking up, the decision to speak up is complicated. It involves individual factors, such as perception of benefit and risk by speaking up or withholding voices, previous positive or negative experience, and sociocultural background, as well as environmental factors represented by the speaking up related climate (psychological safety, encouraging environment, and resignation towards speaking up) in the organization [1, 11]. Studies have been performed worldwide to investigate factors affecting speaking up behavior (perceived concerns, speaking up, and withholding voice) of healthcare workers [1, 2, 8–11, 15]. Different hierarchical levels, professions, and departments were associated with varying degrees of speaking up behavior among healthcare workers.[7–10] However, our understanding of speaking up behavior remains incomplete, and the association between speaking up behavior and other factors, such as the duration of employment and working hours in patient care, has not yet been examined. Furthermore, speaking up behavior and speaking up related climate of healthcare workers in authoritarian societies where healthcare workers in lower levels of authority or different professions hesitate to speak up against those who work at higher levels of authority or varied occupation, such as those in Malaysia, have not yet been explored.

Thus, this cross-sectional study aimed to examine factors associated with the speaking up behavior of healthcare workers and investigate the associations between speaking up behavior and speaking up related climate in a Malaysian hospital. This study will provide an improved understanding of the current situation regarding patient safety in Malaysia and can be used in other countries facing similar challenges.

## Methods

### Aim

This study aimed to determine factors associated with the speaking up behavior of healthcare workers and evaluate the associations between speaking up behavior and the speaking up related climate in a Malaysian hospital.

### Study design and participants

This cross-sectional study was performed at Sarawak General Hospital, which is a tertiary hospital located in East Malaysia, from November 2019 to February 2020. In 2019, Sarawak General Hospital had 1055 beds, 65,518 inpatient admissions, 105,048 Emergency and Trauma Department attendances, and 315,233 outpatients. This is the largest hospital in Sarawak State with a total of 4138 healthcare workers and 448 non-healthcare workers providing almost 43 specialty services and receiving referrals from other district hospitals, specialist hospitals, and private hospitals in the state.

Data were collected from healthcare workers (doctors, nurses, pharmacists, assistant medical officers, assistant pharmacists, radiology technicians, and healthcare assistants) using a self-administered questionnaire as shown below. The sample size was estimated as 384 using Cochran’s formula with a 95% confidence level and 5% margin of error [16]. The estimated response rate was 70%; therefore, 550 hospital healthcare workers were selected for the study. Stratified sampling techniques were used to select 550 samples based on the proportion of the total number of healthcare workers in each department. Of the 550 selected healthcare workers, 474 from 43 departments agreed to participate in the study and answered the questionnaire, resulting in a response rate of 86.2%. All questionnaires were checked for completeness upon returning to the data collector, and there were no missing data. The survey was anonymous and confidentiality was maintained. Written informed consent was obtained from all participants.

### Speaking up for patient safety questionnaire (SUPS-Q)

This study used the SUPS-Q survey instrument developed by the Swiss Patient Safety Foundation, Zurich. Permission to use this survey instrument was obtained from the original author [17]. The instrument (refer to Appendix 1) has two domains. One is speaking up behavior domain with 10 items on three scales (frequency of perceived concerns, speaking up, and withholding voices). The responses were coded in a 5-point Likert scale ranging from “never (0 time)” to “very often (> 10 times).” The other is the speaking up related climate domain with 11 items on three scales (degree of psychological safety for speaking up, encouraging environment for speaking up, and resignation towards speaking up). Responses were coded on a 7-point Likert scale ranging from “strongly disagree” to “strongly agree.” These two domains were used to collect responses in 4 weeks prior to data analysis.

No modifications were made to the content of these two domains in this study. Original survey instrument has two additional sections: perceived barriers to speaking up and the vignette. However, these two sections were not included in this study because they were not related to the aim of this study. The original English version of the survey instrument was used in this study because all respondents understood English.

### Socio-demographic factors and categories

This questionnaire also collected information on the socio-demographic factors of healthcare workers. The socio-demographic factors included sex, age group, profession, department, weekly work hours for patient care, years of employment in the hospital, and hierarchical level. Age was categorized into three groups: 18–35, 35–50, and 50–60 years. Professions were categorized as doctors, nurses, and others (pharmacists, radiology technicians, assistant medical officers, assistant pharmacists, and healthcare assistants). Departments were categorized into medical-based departments, surgical-based departments, emergency and intensive care departments, and others (radiology department, nuclear medicine department, and department of blood transfusion) according to the framework of specialty decided by the Ministry of Health Malaysia [18]. Weekly work hours for patient care were categorized into < 10, 10–24, 24–40, and ≥ 40 h. The years of employment in the hospital were categorized as < 2, 2–5, 5–10, 10–20, and ≥ 20 years. The hierarchical level was categorized as high or low based on whether they had managerial functions, such as the head of a department or unit, nursing matron, nursing sister, or consultant in charge of the ward.

### Statistical analyses

Descriptive statistics (number and percentages) were calculated for the seven variables (sex, age group, profession, department, weekly work hours for patient care, years of employment in the hospital, and hierarchical level). The frequencies of speaking up behavior were scored from one to five: never (score = 1), rarely (score = 2), sometimes (score = 3), often (score = 4), and very often (score = 5). The degrees of speaking up related climate was scored from one to seven: strongly disagree (score = 1), disagree (score = 2), somewhat disagree (score = 3), neutral (score = 4), somewhat agree (score = 5), agree (score = 6), and strongly agree (score = 7). For each study participant, mean scores of the frequencies of speaking up behavior (minimum = 1, maximum = 5) and the degrees of speaking up related climate (minimum = 1, maximum = 7) were calculated for all scales. Higher mean score indicated high frequency of speaking up behavior or strong degree of speaking up related climate. Cronbach’s alpha was calculated as a measure of reliability and internal consistency of scales with values > 0.7 indicating acceptable consistency [19]. Furthermore, means and standard deviations of these mean scores were calculated for all scales according to the seven variables. Analysis of variance (ANOVA) and independent *t* tests were used to determine the statistical differences in the mean scores among the seven variables.

Mean scores of the frequencies of speaking up and withholding voices in each study participant were further classified into dichotomous outcomes using the sample median as the cutoff value [9]; high (frequent speaking up or withholding voices) and low. Univariate and multivariate logistic regression analyses were performed to measure the odds ratio (OR) and adjusted odds ratio (AOR) of frequent speaking up or withholding voices for all seven variables. For multivariate logistic regression analyses, all seven variables were entered to the model. Similarly, mean scores of the degrees of speaking up related climate scales (psychological safety, encouraging environment, and resignation towards speaking up) in each study participant were classified into dichotomous high and low using the sample median as the cutoff value, thereafter, multivariate logistic regression analyses were performed to analyze associations between frequent speaking up or withholding voices and a particular dichotomized speaking up related climate scale. All seven variables mentioned above were entered into the model as covariables; however, other two dichotomized speaking up related climate scales were removed because mean scores of degree of speaking up related climate scales correlated with each other. *P* value < 0.05 was considered statistically significant. Microsoft Excel version 2019 and IBM SPSS version 27 (IBM SPPS Inc., Armonk, NY, USA) were used for statistical analyses.

### Ethical considerations

The study protocol was approved by the Medical Research and Ethics Committee of the National Medical Research Registry, Ministry of Health Malaysia (Approval number: NMRR-19-2042-49000) on August 15, 2019.

## Results

### Socio-demographic characteristics of study participants

Of the 474 study participants, 83.5% (*n =* 396) were female, 66.4% (*n =* 315) were between 18 and 35 years of age, and 75.5% (*n =* 358) were nurses (Table 1). Most respondents were from surgical-based departments (42.8%, *n =* 203), followed by medical-based departments (38.4%, *n =* 182), emergency and intensive care departments (15.6%, *n =* 74), and others (3.2%, *n =* 15). More than half of the respondents worked in patient care for ≥ 40 h per week (54.9%, *n =* 260). Most respondents had been employed for 5–10 years in the hospital (34.0%, *n =* 144), followed by those employed for 10–20 years (30.4%, *n =* 144). The majority (85.0%, *n =* 403) were categorized as having a low hierarchical level.

**Table 1:** Socio-demographic characteristics of the study population

Variables	Total (*N* = 474)	
	*N*	(%)
Sex		
Male	78	(16.5)
Female	396	(83.5)
Age group (years)		
18–35	315	(66.4)
35–50	144	(30.4)
50–60	15	(3.2)
Profession		
Doctor	92	(19.4)
Nurse	358	(75.5)
Other^a^	24	(5.1)
Department		
Medical-based	182	(38.4)
Surgical-based	203	(42.8)
Emergency and intensive care	74	(15.6)
Other^b^	15	(3.2)
Weekly work hours for patient care		
< 10 h	59	(12.4)
10–24 h	70	(14.8)
24–40 h	85	(17.9)
≥ 40 h	260	(54.9)
Years of employment in the hospital		
< 2 years	36	(7.6)
2–5 years	84	(17.7)
5–10 years	161	(34.0)
10–20 years	144	(30.4)
≥ 20 years	49	(10.3)
Hierarchical level^c^		
Low	403	(85.0)
High	71	(15.0)

^a^Other includes pharmacists, radiology technicians, assistant medical officers, assistant pharmacists, and healthcare assistants

^b^Other includes radiology department, nuclear medicine department, and department of blood transfusion

^c^The high level indicates positions with managerial functions, such as the head of department or unit, nursing matron, nursing sister, or consultant in charge of the ward. The other positions are categorized as low level

### Speaking up behavior and speaking up related climate

The mean scores for the frequencies of speaking up behavior (perceived concerns, speaking up, and withholding voices) and the degrees of speaking up related climate (psychological safety, encouraging environment, and resignation towards speaking up) according to sex, age group, profession, department, weekly work hours for patient care, years of employment in the hospital, and hierarchical level are presented in Table 2.

**Table 2:** Frequency of speaking up behavior and degree of speaking up related climate (*N =* 474)

Variables	Frequency of speaking up behavior						Degree of speaking up related climate					
	Perceived concerns (Cronbach’s α = 0.62)		Speaking up (Cronbach’s α = 0.70)		Withholding voices (Cronbach’s α = 0.84)		Psychological safety (Cronbach’s α = 0.89)		Encouraging environment (Cronbach’s α = 0.92)		Resignation towards speaking up (Cronbach’s α = 0.89)	
	Mean (SD)^a^	*P* value^c^	Mean (SD)^a^	*P* value^c^	Mean (SD)^a^	*P* value^c^	Mean (SD)^b^	*P* value^c^	Mean (SD)^b^	*P* value^c^	Mean (SD)^b^	*P* Value^c^
Total	3.24 (0.76)		3.44 (0.78)		2.22 (0.76)		5.16 (1.08)		5.21 (1.11)		4.14 (1.58)	
Sex		0.019		0.404		0.505		0.018		0.972		0.840
Male	3.06 (0.81)		3.38 (0.83)		2.17 (0.81)		5.42 (1.17)		5.21 (1.24)		4.17 (1.83)	
Female	3.28 (0.75)		3.46 (0.77)		2.23 (0.75)		5.10 (1.06)		5.20 (1.08)		4.13 (1.53)	
Age group (years)		0.009		0.116		0.420		0.483		0.555		0.980
18–35	3.16 (0.76)		3.39 (0.76)		2.24 (0.73)		5.19 (1.10)		5.16 (1.14)		4.15 (1.62)	
35–50	3.39 (0.77)		3.55 (0.82)		2.18 (0.81)		5.07 (1.05)		5.27 (1.08)		4.12 (1.54)	
50–60	3.42 (0.62)		3.57 (0.64)		2.02 (0.69)		5.17 (0.99)		5.36 (0.78)		4.16 (1.26)	
Profession		< 0.001		< 0.001		0.155		0.745		0.501		0.070
Doctor	2.76 (0.86)		3.10 (0.78)		2.10 (0.82)		5.21 (1.10)		5.08 (1.18)		4.45 (1.56)	
Nurse	3.36 (0.68)		3.53 (0.76)		2.25 (0.74)		5.15 (1.08)		5.24 (1.09)		4.05 (1.60)	
Other^d^	3.35 (0.83)		3.41 (0.72)		2.21 (0.70)		5.11 (1.04)		5.18 (1.09)		4.19 (1.26)	
Department		0.044		< 0.001		0.037		0.001		0.001		0.003
Medical-based	3.34 (0.64)		3.57 (0.76)		2.25 (0.73)		5.22 (1.06)		5.21 (1.03)		3.95 (1.57)	
Surgical-based	3.19 (0.85)		3.46 (0.78)		2.18 (0.75)		5.18 (1.02)		5.30 (1.09)		4.28 (1.54)	
Emergency and intensive care	3.19 (0.77)		3.16 (0.77)		2.34 (0.78)		4.80 (1.18)		4.80 (1.18)		4.44 (1.54)	
Other^e^	2.91 (0.73)		3.03 (0.63)		1.73 (0.83)		5.89 (1.33)		5.91 (1.42)		3.09 (1.99)	
Weekly work hours for patient care		0.101		0.003		0.427		0.372		0.352		0.057
< 10 h	3.38 (0.73)		3.76 (0.83)		2.14 (0.78)		5.22 (1.23)		5.24 (1.13)		4.07 (1.63)	
10–24 h	3.35 (0.65)		3.40 (0.79)		2.29 (0.84)		5.08 (1.18)		5.03 (1.28)		4.35 (1.36)	
24–40 h	3.28 (0.77)		3.51 (0.68)		2.13 (0.69)		4.99 (1.13)		5.12 (1.12)		3.74 (1.48)	
≥ 40 h	3.17 (0.79)		3.36 (0.78)		2.24 (0.75)		5.21 (1.00)		5.27 (1.06)		4.22 (1.65)	
Years of employment in the hospital		0.012		0.007		0.004		0.739		0.243		0.813
< 2 years	2.72 (0.93)		3.00 (0.96)		2.01 (0.90)		5.26 (1.12)		5.18 (1.27)		4.22 (1.61)	
2–5 years	3.12 (0.77)		3.37 (0.71)		2.36 (0.67)		5.14 (1.06)		5.04 (1.09)		4.10 (1.65)	
5–10 years	3.26 (0.68)		3.40 (0.78)		2.25 (0.72)		5.21 (1.13)		5.21 (1.12)		4.18 (1.66)	
10–20 years	3.37 (0.78)		3.56 (0.75)		2.27 (0.81)		5.06 (1.07)		5.17 (1.12)		4.04 (1.49)	
≥ 20 years	3.38 (0.68)		3.67 (0.71)		1.87 (0.62)		5.23 (0.99)		5.59 (0.88)		4.31 (1.47)	
Hierarchical level^f^		0.004		0.005		0.138		0.371		0.779		0.003
Low	3.18 (0.77)		3.39 (0.78)		2.25 (0.72)		5.17 (1.08)		5.18 (1.11)		4.06 (1.58)	
High	3.58 (0.66)		3.76 (0.70)		2.04 (0.94)		5.06 (1.13)		5.34 (1.11)		4.55 (1.53)	

^a^Mean score: minimum = 1, maximum = 5. ^b^Mean score: minimum = 1, maximum = 7

^c^*P* value is determined based on analysis of variance and independent *t* test

^d^Other includes pharmacists, radiology technicians, assistant medical officers, assistant pharmacists, and healthcare assistants

^e^Other includes radiology department, nuclear medicine department, and department of blood transfusion

^f^The high level includes positions with managerial functions, such as the head of department or unit, nursing matron, nursing sister, or consultant in charge of the ward. The other positions were categorized as low level

For the frequencies of speaking up behavior, the Cronbach’s alpha values for perceived concerns, speaking up, and withholding voices were 0.62, 0.70, and 0.84, respectively, indicating acceptable internal consistency. Nurses had higher mean scores for perceived concerns, speaking up, and withholding voices than doctors. Healthcare workers in emergency and intensive care departments showed a higher mean score for withholding voices than those in medical-based, surgical-based, and other departments, which also showed a lower mean score for speaking up. The mean scores for perceived concerns and speaking up increased based on the duration of employment and decreased based on the weekly work hours for patient care. Healthcare workers with 2–5 years of employment reported the highest mean score for withholding voices. Healthcare workers with high hierarchical levels were found to perceive more concerns, speak up more frequently than their subordinates, and were less likely to withhold their voices.

Regarding speaking up related climate, the Cronbach’s alpha values for psychological safety, encouraging environment, and resignation towards speaking up were 0.89, 0.92, and 0.89, respectively, indicating acceptable internal consistency. Healthcare workers in emergency and intensive care departments showed the lowest mean score for psychological safety and encouraging environment and the highest mean score for resignation towards speaking up. Healthcare workers with a high hierarchical level demonstrated a significantly higher mean score for resignation towards speaking up than their counterparts. Healthcare workers with 2–5 years of employment had relatively low mean scores for psychological safety and encouraging environment.

### Bivariate analysis of factors associated with frequent speaking up

Univariate and multivariate analysis results for frequently speaking up are shown in Table 3. Nurses were approximately twice more likely to speak up than doctors (AOR 2.07, 95% CI 1.01–4.05). Emergency and intensive care department healthcare workers had fewer experiences of speaking up than those in medical-based departments (AOR 0.45, 95% CI 0.25–0.81). Healthcare workers who work ≥ 40 h per week were approximately half as likely to speak up compared to workers who work < 10 h (AOR 0.47, 95% CI 0.24–0.92). Healthcare workers with an employment period of 10–20 years and those with an employment period of ≥ 20 years were approximately three times more likely to speak up than those having < 2 years of employment (AOR 2.78, 95% CI 1.28–6.01 and AOR 2.82, 95% CI 1.16–7.04). Healthcare workers with a high hierarchical level were 2.7 times more likely to speak up than their lower hierarchical counterparts. (AOR 2.70, 95% CI 1.32–5.52).

**Table 3:** Univariate and multivariate analyses of frequent speaking up (*N =* 474)

Variables	Frequency of speaking up^a^				OR	95% CI	*P* value	AOR^b^	95% CI	*P* value
	High (*N =* 259)		Low (*N =* 215)							
	N	(%)	N	(%)						
Sex										
Male	41	(15.8)	37	(17.2)	1	Reference		1	Reference	
Female	218	(84.2)	178	(82.8)	1.11	0.68–.1.80	0.687	1.53	0.83–2.84	0.176
Age group (years)										
18–35	164	(63.3)	151	(70.2)	1	Reference		1	Reference	
35–50	87	(33.6)	57	(26.5)	1.41	0.94–2.10	0.096	1.32	0.88–2.00	0.184
50–60	8	(3)	7	(3.3)	1.05	0.37–2.97	0.923	0.97	0.34–2.81	0.971
Profession										
Doctor	35	(13.5)	57	(26.5)	1	Reference		1	Reference	
Nurse	209	(80.7)	149	(69.3)	2.28	1.43–3.66	0.001	2.07	1.01–4.05	0.032
Other^c^	15	(5.8)	9	(4.2)	2.71	1.07–6.86	0.035	3.03	0.99–9.21	0.051
Department										
Medical-based	111	(42.9)	71	(33.0)	1	Reference		1	Reference	
Surgical-based	116	(44.8)	87	(40.5)	0.85	0.57–1.28	0.444	0.88	0.56–1.36	0.557
Emergency and intensive care	28	(10.8)	46	(21.4)	0.39	0.22–0.68	0.001	0.45	0.25–0.81	0.008
Other^d^	4	(1.5)	11	(5.1)	0.23	0.07–0.76	0.016	0.14	0.04–0.51	0.003
Weekly work hours for patient care										
< 10 h	42	(16.2)	17	(7.9)	1	Reference		1	Reference	
10–24 h	38	(14.7)	32	(14.9)	0.48	0.23–1.00	0.050	0.59	0.27–1.28	0.183
24–40 h	53	(20.5)	32	(14.9)	0.67	0.33–1.37	0.272	0.65	0.30–1.40	0.270
≥ 40 h	126	(48.6)	134	(62.3)	0.38	0.21–0.70	0.002	0.47	0.24–0.92	0.027
Years of employment in the hospital										
< 2 years	14	(5.4)	22	(10.2)	1	Reference		1	Reference	
2–5 years	40	(15.4)	44	(20.5)	1.43	0.65–3.16	0.379	1.44	0.65–3.19	0.372
5–10 years	84	(32.4)	77	(35.8)	1.71	0.82–3.59	0.152	1.79	0.84–3.79	0.129
10–20 years	90	(34.8)	54	(25.1)	2.62	1.24–5.55	0.012	2.78	1.28–6.01	0.010
≥ 20 years	31	(12.0)	18	(8.4)	2.71	1.12–6.57	0.028	2.82	1.16–7.04	0.023
Hierarchical level^e^										
Low	208	(80.3)	195	(90.7)	1	Reference		1	Reference	
High	51	(19.7)	20	(9.3)	2.39	1.38–4.16	0.002	2.70	1.32–5.52	0.006

^a^Sample median of speaking up (median = 3.50) was used as the cutoff value

^b^Adjusted odds ratio using all other variables as covariates

^c^Other includes pharmacists, radiology technicians, assistant medical officers, assistant pharmacists, and healthcare assistants

^d^Other includes radiology department, nuclear medicine department, and department of blood transfusion

^e^The high level indicates positions with managerial functions, such as the head of department or unit, nursing matron, nursing sister, or consultant in charge of the ward. The other positions are categorized as low level

OR, odds ratio; AOR, adjusted odds ratio, 95% CI, 95% confident interval

### Bivariate analysis of factors associated with frequent withholding voices

Univariate and multivariate analysis results for frequent withholding voices are presented in Table 4. Univariate logistic regression analyses indicated healthcare workers from surgical-based departments and those with high hierarchical levels were less likely to withhold their voices compared to those from medical-based departments and those with low hierarchical levels. Compared to those having < 2 years of employment, healthcare workers with an employment period of 2–5 years were 2.2 times more likely to withhold their voices. However, these differences were not statistically significant as per multivariate logistic regression analyses.

**Table 4:** Univariate and multivariate analyses of frequent withholding voices (*N =* 474)

Variables	Frequency of withholding voices^a^				OR	95% CI	*P* value	AOR^b^	95% CI	*P* value
	High (*N =* 255)		Low (*N =* 219)							
	N	(%)	N	(%)						
Sex										
Male	37	(14.5)	41	(18.7)	1	Reference		1	Reference	
Female	218	(85.5)	178	(81.3)	1.36	0.83–2.21	0.219	1.28	0.71–2.33	0.405
Age group (years)										
18–35	176	(69.0)	139	(63.5)	1	Reference		1	Reference	
35–50	72	(28.2)	72	(32.9)	0.79	0.53–1.17	0.242	0.28	0.07–1.17	0.081
50–60	7	(2.7)	8	(3.7)	0.69	0.25–1.95	0.486	0.41	0.11–1.45	0.171
Profession										
Doctor	43	(16.9)	49	(22.4)	1	Reference		1	Reference	
Nurse	199	(78.0)	159	(72.6)	1.43	0.90–2.26	0.130	1.85	0.96–3.56	0.066
Other^c^	13	(5.1)	11	(5.0)	1.35	0.55–3.32	0.517	2.24	0.79–6.36	0.130
Department										
Medical-based	107	(42.0)	75	(34.2)	1	Reference		1	Reference	
Surgical-based	98	(38.4)	105	(48.0)	0.65	0.44–0.98	0.039	0.76	0.50–1.18	0.222
Emergency and intensive care	45	(17.6)	29	(13.2)	1.09	0.63–1.89	0.766	1.16	0.65–2.10	0.615
Other^d^	5	(2.0)	10	(4.6)	0.35	0.12–1.07	0.065	0.34	0.11–1.11	0.073
Weekly work hours for patient care										
< 10 h	29	(11.4)	30	(13.7)	1	Reference		1	Reference	
10–24 h	38	(14.9)	32	(14.6)	1.23	0.61–2.46	0.561	1.12	0.54–2.33	0.755
24–40 h	41	(16.1)	44	(20.1)	0.96	0.49–1.87	0.914	0.96	0.47–1.95	0.908
≥ 40 h	147	(57.6)	113	(51.6)	1.35	0.76–2.37	0.304	1.32	0.71–2.45	0.379
Years of employment in the hospital										
< 2 years	17	(6.7)	19	(8.7)	1	Reference		1	Reference	
2–5 years	56	(22.0)	28	(12.8)	2.24	1.01–4.96	0.048	2.21	0.98–4.93	0.054
5–10 years	88	(34.5)	73	(33.3)	1.35	0.65–2.78	0.420	1.19	0.57–2.51	0.640
10–20 years	79	(31.0)	65	(29.7)	1.36	0.65–2.82	0.412	1.02	0.43–2.39	0.965
≥ 20 years	15	(5.8)	34	(15.5)	0.49	0.20–1.20	0.121	0.28	0.09–0.88	0.129
Hierarchical level^e^										
Low	229	(89.8)	174	(79.6)	1	Reference		1	Reference	
High	26	(10.2)	45	(20.5)	0.44	0.26–0.74	0.002	0.61	0.31–1.18	0.139

^a^Sample median of withholding voices (median = 2.25) was used as the cutoff value

^b^Adjusted odds ratio using other variables as covariates

^c^Other includes pharmacists, radiographers, assistant medical officers, assistant pharmacists, and healthcare assistants

^d^Other includes radiology department, nuclear medicine department, and department of blood transfusion

^e^The high level indicates positions with managerial functions, such as the head of department or unit, nursing matron, nursing sister, or consultant in charge of the ward. The other positions are categorized as low level

OR, odds ratio; AOR, adjusted odds ratio, 95 CI,  95 confidence interval

### Association between speaking up behavior and speaking up related climate

Multivariate logistic regression analysis between speaking up behavior and speaking up related climate are shown in Table 5. In an encouraging environment, healthcare workers were more likely to speak up (AOR 2.19, 95% CI 1.43–3.36) and less likely to withhold their voices (AOR 0.54, 95% CI 0.36–0.83). Healthcare workers in a discouraging environment which inhibited their speaking up ability were more likely to withhold their voices (AOR 4.74, 95% CI 3.07–7.31).

**Table 5:** Association between speaking up behavior and speaking up related climate

Variables	Frequent speaking up^a^		Frequent withholding voices^b^	
	AOR (95% CI)^c^	*P* values	AOR (95% CI)^c^	*P* values
Psychological safety^d^				
Low	1 (Reference)		1 (Reference)	
High	1.46 (0.99–2.16)	0.058	0.69 (0.47–1.01)	0.056
Encouraging environment^e^				
Low	1 (Reference)		1 (Reference)	
High	2.19 (1.43–3.36)	< 0.001	0.54 (0.36–0.83)	0.005
Resignation towards speaking up^f^				
Low	1 (Reference)		1 (Reference)	
High	1.07 (0.72–1.61)	0.740	4.74 (3.07–7.31)	< 0.001

^a^Sample median of speaking up was used as the cutoff value (median = 3.50)

^b^Sample median of withholding voices was used as the cutoff value (median = 2.25)

^c^Adjusted odds ratio using sex, age group, profession, department, weekly work hours for patient care, years of employment in the hospital and hierarchical level as covariates

^d^Sample median of psychological safety was used as the cutoff value (median = 5.20)

^e^Sample median of encouraging environment was used as the cutoff value (median = 5.00)

^f^Sample median of resignation towards speaking up was used as the cutoff value (median = 4.00)

AOR, adjusted odds ratio; 95% CI, 95% confidence interval

## Discussion

Limited studies been published on patient safety culture in Malaysian hospitals [20–23]. To our knowledge, this is the first study to examine the factors associated with speaking up behavior and speaking up related climate among healthcare workers in Malaysia. This study revealed that healthcare workers, who worked in emergency and intensive care departments, had long weekly working hours for patient care, of short employment duration, and at low hierarchical levels were less likely to speak up. A strong association between an encouraging environment and frequent speaking up was observed. Higher levels of resignation towards speaking up were strongly associated with higher levels of withholding the voice.

We found that doctors were lesser inclined to speak up than nurses, which was similar to the results of previous studies [8–10]. A study performed in Switzerland revealed that doctors were reluctant to speak up when the clinical situation was under unclear risk [10]. A qualitative study in a Hong Kong hospital revealed that doctors would speak up only when their opinion and justification were strong [15]. Accordingly, doctors often perceived speaking up as a risk with a fear of shame or repercussion, especially when the situation was uncertain or ambiguous [24, 25]. In this study, high frequencies of withholding voices and speaking up, based on high frequencies of perceived concerns, among nurses showed that both behaviors frequently coexisted and were not opposed completely.

Healthcare workers from the emergency and intensive care departments had significantly fewer experiences of speaking up than those from medical-based departments. This was also supported by high levels of withholding voices, low levels of psychological safety, a less encouraging environment, and high resignation towards speaking up in emergency and intensive care departments. Healthcare workers in these departments may face barriers for speaking up due to heavy workloads and interdisciplinary collaboration [26]. These findings are of great concern because these two departments are at high risk of medical error due to their fast pace, frequent interruptions, and multiple handovers, which lead to severe patent harm [26]. Therefore, establishing a culture of speaking up is crucial, and policies and procedures for expressing safety concerns should be strengthened in these departments [27].

This study also found that the mean score for speaking up among healthcare workers increased with the duration of employment. This finding is also consistent with higher levels of perceived concern with a longer duration of employment. These findings indicate that compliance to patient safety has been developed with experiences of healthcare. Compared to their junior and senior counterparts, healthcare workers with 2–5 years of employment reported the highest level of withholding voice. Healthcare workers with 2–5 years of employment are considered intermediate hierarchical levels and are usually those who have finished their probation or internship period. Therefore, they may perceive that withholding voices could be a safe option due to fear of punishment or concerns regarding incompetence [28].

Compared to those having short working hours, healthcare workers with long working hours in patient care were less likely to speak up, although healthcare workers with long working hours were expected to speak up more often because they may have had more chances to encounter medical errors than those with short working hours. Nurses who were too busy and suffering from burnout did not speak up [29]. An association between burnout and withholding voices among healthcare workers was previously observed [30]. Long working hours and high workloads caused mental and physical fatigue in healthcare workers, consequently reducing their ability to recognize safety concerns and speak up.

Healthcare workers with high hierarchical levels were found to speak up more than those with low hierarchical levels. These results are consistent with those of the studies conducted in Asian countries, such as Japan, Hong Kong, South Korea, and Taiwan [15, 26, 31]. Healthcare workers with low hierarchies may perceive that their voices are not valued, leading to reluctance to speak up. However, studies performed in Austria and Switzerland revealed that healthcare workers with low hierarchies were found to speak up more often [12, 14]. These results suggest that the patient safety culture may differ between Western and Asian countries and that the authoritarian culture may be stronger in Asian than in Western countries [32, 33].

However, in this study, healthcare workers with high hierarchical levels showed higher resignations towards speaking up than their counterparts. This seems to contradict the finding that they also spoke up relatively frequently. These findings demonstrate the ambivalent attitude and struggle for speaking up among healthcare workers with managerial functions. They recognize the importance of speaking up and frequently move in action while also experience frustration and resignation towards speaking up.

This study has some limitations. First, it was performed at a single institution; therefore, the findings may not be representative of the situation in Malaysia as a whole. However, enough sample size and designed sampling methods in the present study might assure external validity to some extent. Further research involving multiple institutions is required to improve the external validity. Second, a causal relationship could not be established because of the cross-sectional nature of the study. Third, there is a possibility of recall bias because this study used a self-administered questionnaire that assessed the experiences in 4 weeks prior to data analysis. Finally, this study did not evaluate the participants’ negative experiences related to speaking up, their workload, and possession of specialties, which may have influenced their actions.

## Conclusions

This study provides robust evidence on the current situation regarding patient safety in Malaysia indicating that healthcare workers in emergency and intensive care department, those with short years of employment, and those who worked at low hierarchical levels were less likely to speak up. This study also illustrated a strong association between frequent speaking up and an encouraging environment, as well as between frequent withholding of voices and a discouraging environment. It is essential to establish an encouraging environment that promotes speaking up and prevents withholding voices among healthcare workers to ensure patient safety and prevent harm, especially in circumstances where multiple types of healthcare workers with different socio-demographic backgrounds work together.

## Data Availability

The data sets used and/or analyzed during the current study are available from the corresponding author on reasonable request.

## Appendix 1

**Table:**

Frequency of speaking up behavior
Over the last 4 weeks
Never (0 time)/Rarely (1–2 times)/Sometimes (3–5 times)/Often (6–10 times)/Very often (> 10 times)
*Perceived concerns*
1. How often have you had specific concerns about patient safety?
2. How often have you observed an error that, if uncaptured, could be harmful to patients?
3. How often have you noticed that your colleagues haven’t followed important patient safety rules, intentionally or unintentionally?
*Speaking up*
4. How many times did you bring up specific concerns about patient safety?
5. How many times did you address an error that, if uncaptured, could be harmful to patients?
6. How many times did you address a colleague (doctors and/or nurses) when he/she didn’t follow important patient safety rules, intentionally or unintentionally?
7. How many times did you prevent an incident from occurring because of bringing up specific concerns about patient safety?
*Withholding voices*
8. How many times did you choose not to bring up your specific concerns about patient safety?
9. How many times did you keep ideas for improving patient safety in your unit to yourself?
10. How many times did you remain silent when you had information that might have prevented a safety incident in your unit?

Degree of speaking up related climate
Strongly disagree/disagree/somewhat disagree/neutral/somewhat agree/agree/strongly agree
*Psychological safety for speaking up*
1. I can rely on my colleagues (doctors and/or nurses), whenever I encounter difficulties in my work
2. I can rely on the shift supervisor (person in charge of a shift) whenever I encounter difficulties in my work
3. The culture in my unit/clinical area makes it easy to speak up about patient safety concerns
4. My colleagues (doctors and/or nurses) react appropriately when I speak up about my concerns about patient safety
5. My shift supervisors (person in charge) react appropriately when I speak up about my patient safety concerns
*Encouraging environment for speaking up*
6. In my unit/clinical area, I observe others speaking up about their patient safety concerns
7. I am encouraged by my colleagues (doctors and/or nurses) to speak up about patient safety concerns
8. I am encouraged by my shift supervisor (the person in charge during a shift) to speak up about patient safety concerns
*Resignation towards speaking up*
9. Having to remind staff of the same safety rules, again and again, is frustrating
10. Sometimes I become discouraged because nothing changes after expressing my patient safety concerns
11. When I have concerns regarding patient safety, it is difficult to submit them

Speaking up for patient safety questionnaire (SUPS-Q)–speaking up behavior domain and speaking up related climate domain)

## Abbreviations

OR: Odds ratio
SUPS-Q: Speaking up for patient safety questionnaire
ANOVA: Analysis of variance
